# Phenytoin Inhibits the Persistent Sodium Current in Neocortical Neurons by Modifying Its Inactivation Properties

**DOI:** 10.1371/journal.pone.0055329

**Published:** 2013-01-29

**Authors:** Elisa Colombo, Silvana Franceschetti, Giuliano Avanzini, Massimo Mantegazza

**Affiliations:** 1 Department of Neurophysiopathology – Epilepsy Center, Foundation IRCCS Neurological Institute C. Besta, Milan, Italy; 2 Institut de Pharmacologie Moléculaire et Cellulaire (IPMC), CNRS UMR7275 and University of Nice-Sophia Antipolis, Valbonne, France; McGill University, Canada

## Abstract

The persistent Na^+^ current (I_NaP_) is important for neuronal functions and can play a role in several pathologies, although it is small compared to the transient Na^+^ current (I_NaT_). Notably, I_NaP_ is not a real persistent current because it undergoes inactivation with kinetics in the order of tens of seconds, but this property has often been overlooked. Na^+^ channel blockers, drugs used for treating epilepsy and other diseases, can inhibit I_NaP_, but the mechanism of this action and the conditions in which I_NaP_ can be actually inhibited have not been completely clarified yet. We evaluated the action of phenytoin (PHT), a prototype anti-epileptic Na^+^ channel blocker, on I_NaP_ inactivation in pyramidal neurons of rat sensorimotor cortical slices at different concentrations, from 5 to 100 µM. PHT did not modify I_NaP_ evoked with depolarizing voltage ramps of 50 or 100 mVs^−1^, but decreased I_NaP_ evoked by slower voltage ramps (10 mVs^−1^). However, at all of the tested concentrations, PHT decreased I_NaP_ evoked by faster ramps when they were preceded by inactivating pre-pulses. Moreover, PHT shifted towards negative potentials the voltage-dependence of I_NaP_ inactivation and accelerated its kinetics of development also at depolarized potentials (+40 mV), not consistently with a simple inactivated state stabilizer. Therefore, our study shows a prominent PHT effect on I_NaP_ inactivation rather than an open channel block, which is instead often implied. I_NaP_ is inhibited by PHT only in conditions that induce major I_NaP_ inactivation. These results highlight the importance of I_NaP_ inactivation not only for physiological functions but also as drug target, which could be shared by other therapeutic drugs. Through this action PHT can reduce I_NaP_-induced long-lasting pathological depolarisations and intracellular sodium overload, whereas shorter I_NaP_ actions should not be modified. These properties set the conditions of efficacy and the limits of PHT as I_NaP_ inhibitor.

## Introduction

A small fraction of the tetrodotoxin (TTX)-sensitive voltage-dependent Na^+^ current has been defined “persistent” (I_NaP_) because it flows after the classical transient Na^+^ current (I_NaT_) has undergone inactivation [1], [2]. I_NaP_ has been observed in several excitable cells. In the central nervous system, it was initially characterized in cortical neurons [1]. Despite its small amplitude compared with the peak of I_NaT_, it can influence the properties of neuronal excitability because it begins activating in the sub-threshold voltage range and flows throughout repetitive neuronal discharges. In fact, I_NaP_ can contribute to shaping firing characteristics, boosting synaptic inputs, generating sub-threshold membrane oscillations, sustaining pacemaking and maintaining long depolarized plateau potentials in many neuron types [1], [3]–[9]. In particular, it has been shown that I_NaP_ plays an important role in the proximal axon of cortical neurons, where synaptic inputs undergo a final integration and action potentials are generated [9]–[12]. Moreover, its properties can be modulated [13]–[17]. Importantly, despite its name, I_NaP_ is not a truly “persistent” current, because it undergoes a process of inactivation with kinetics in the order of tens of seconds. This property can be important for its functions, but it has been characterized only in few studies [4], [7], [8], [18], [19] and often not considered at all; in fact, in most of the studies I_NaP_ has been considered a non-inactivating current [1], [2].

In addition to its physiological role, I_NaP_ can play a role in pathological conditions. In fact, it has been found to be significantly increased in pathologies of the nervous system, in which it can induce neuronal hyperexcitability and/or Na^+^ overload leading to neurodegeneration [2], [20], [21]. In fact, increased I_NaP_ has been implicated both in acquired [22]–[25] and possibly in genetically determined epilepsy [26], [27], [28], but see [29], [30], as well as in familial hemiplegic migraine [31] and in neurodegeneration induced by different types of insults [20], [32], [33], [34].

Because of its role in sustaining epileptic discharges and long membrane depolarizations, I_NaP_ has been repeatedly evaluated as a target of anti-epileptic and neuroprotective drugs [21]. Various reports have shown that it can be reduced by a number of traditional and new anticonvulsant, antiarrhythmic and anesthetic drugs [35]–[42], which are supposed to bind to a common receptor area on Na^+^ channels [21], [43]. However, little is known about the mechanism of reduction of I_NaP_, although a block of channels in the open conformation is frequently implied, because I_NaP_ has often been considered non-inactivating [35], [37]–[42].

We have studied the action of phenytoin (PHT, a prototype anticonvulsant Na^+^ channel blocker) on I_NaP_ recorded in layer V or layer II/III pyramidal neurons in somatosensory cortical slices, showing that PHT selectively acts on I_NaP_ inactivation.

## Materials and Methods

### Slice preparation

All of the experimental procedures were carried out in compliance with the 86/609/UE directive of 14 November 1986 on animal research and the guidelines for animal care and management of the Ethics Committee of the Besta Neurological Institute and of the Italian Ministry of Health, which approved the experimental protocol (Permit Number: SNE 01–10). All efforts were made to minimize the number of animals used, avoid their suffering and follow the Three Rs principle: reduce, refine or replace animal experimentation. Sprague-Dawley rats (Charles River, Italy) aged 13–17 days were anesthetized with isoflurane, decapitated, and their brains were removed and placed in ice-cold artificial cerebrospinal fluid (standard ACSF: 129 mM NaCl, 21 mM NaHCO_3_, 1.6 mM CaCl_2_, 1.25 mM NaH_2_PO_4_, 1.8 mM MgSO_4_, 3 mM KCl and 10 mM glucose), bubbled with 95% O_2_, 5% CO_2_. Coronal slices (300 μm) were prepared from the sensorimotor cortex using a vibratome and transferred to a brain slice pre-chamber kept at room temperature and containing standard ACSF (see above). After a 1 h recovery period, the slices were transferred to a submersion chamber kept at 33°C, and perfused with the external recording solution (see below).

### Electrophysiological recordings

Whole cell patch-clamp recordings were made in layer II/III and V at 33°C using an Axopatch 200B amplifier (MDS/Axon Instr.). Pyramidal neurons were directly visualized in brain slices by means of infrared differential-interference contrast microscopy using an upright microscope (Zeiss Axioscope) and a CCD camera (Hamamatsu).

**Figure 1:**
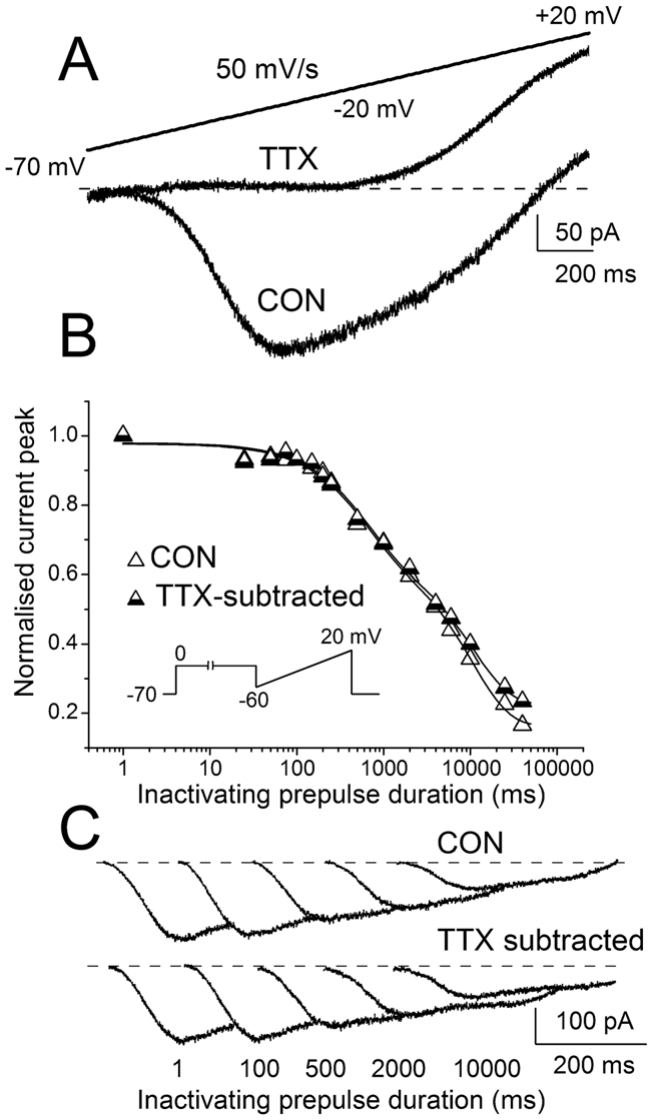
I_NaP_ activation and time dependent inactivation. A, I_NaP_ evoked in a representative neuron by means of a slow voltage ramp compared with the current trace evoked in the presence of TTX, revealing a small outward current activating at more positive potentials. B, kinetics of development of I_NaP_ time-dependent inactivation assessed with and without TTX subtraction, which completely overlap. The data points are fit with a bi-exponential function. The inset illustrates the stimulus protocol. C, examples of I_NaP_ traces with or without TTX subtraction evoked after inactivating prepulses to 0 mV lasting from 100 ms to 10 seconds. The traces are shown partially overlapped to better compare peak amplitude.

The neurons were bath-perfused with an external solution containing 60 mM NaCl, 60 mM TEA-Cl, 0.2 mM CaCl_ 2_, 7 mM MgCl_2_, 2 mM CoCl_2_, 24 mM NaHCO_3_ and 10 mM glucose, and bubbled with 95% O_2_, 5% CO_2_. In some control experiments, TTX 1 µM was added to the perfusing medium in order to rule out the contribution of contaminating currents that could activate in the voltage range used to study I_NaP_ properties (Figure 1). Electrodes were filled with a solution containing 132 mM CsCl, 2 mM MgCl_2_, 10 mM EGTA, 10 mM HEPES and 2 mM Na_2_-ATP, pH 7.25 with CsOH.

Stimuli were generated and signals acquired using a Digidata 1320 digitizer (MDS/Axon Instr.), with pClamp 8.0 software (MDS/Axon Instr.). After seal formation and cell membrane rupturing, capacitance currents were minimized using the amplifier circuitry and 75–80% series resistance compensation was routinely applied. The sampling frequency was 5 kHz for the ramp protocols and 10 kHz for the step protocols; signals were filtered at 1 kHz (voltage ramps) or 5 kHz (step). The recordings with voltage-clamp and space-clamp errors (i.e. the presence of unclamped action currents) were excluded from the analysis. The junction potential error was not corrected.

I_NaP_ was evoked using slow voltage ramps that depolarized the membrane from −70 to +20 mV, with a slope of 50 mV s^−1^ and a total duration of 1.8 s (“standard voltage ramp”) from a holding potential of −70 mV. In some experiments slower (10 mV s^−1^; duration 9 s) or faster (100 mV s^−1^; duration 0.9 s) voltage ramps were also applied. The leak was subtracted from the current traces elicited with voltage ramps with the “subtract slope” within the “baseline adjustment” of pClamp, thus by subtracting the linear fit of the trace performed at potentials more negative than the activation of I_NaP_. For some cells this procedure was compared with the subtraction of the trace recorded in the presence of TTX (see results and [19], showing that the fit-subtraction was faithful (the actual leak was linear) at least up to the peak of I_NaP_. If the leak were constant during the recordings (which in our case are quite long) TTX-subtraction would be the best method, but even small changes can produce significant errors and, moreover, if the cell is lost before the application of TTX it is not possible to leak subtract in this way. Thus, we think that for these recordings it is better to use the linear regression subtraction.

The voltage-dependence of I_NaP_ activation (conductance–voltage, *g*–*V*, curve) was calculated from the currents recorded using voltage ramps as *g* = I_Na_/(*V* – E_Na_), where I_Na_ is the recorded Na^+^ current measured at potential *V* and E_Na_ is the calculated Nernst equilibrium potential. The voltage-dependence of I_NaP_ steady-state inactivation was evaluated by delivering standard voltage ramps at the end of a 10 s depolarizing pulse at voltages ranging from −90 to 0 mV. The time-dependence of inactivation was evaluated by applying depolarizing prepulses to 0 mV lasting from 0 to 40 s and eliciting I_NaP_ by means of a standard voltage ramp. The holding potential was −70 mV and the same potential was maintained for 45 s between the stimuli of every stimulation protocol in order to avoid accumulation of slow inactivation.

In some experiments, we evaluated the effect of PHT on neurons recorded in current-clamp configuration using the I_CLAMP_-FAST setting of the Axopatch 200B, which can reliably record action potentials in large neurons [44]. The effect of 100 µM PHT was assessed by measuring the duration and decay of the depolarized plateau that typically follow action potentials after that Ca^2+^ and K^+^ currents are blocked, and which are assumed to be substantially sustained by I_NaP_ [3]. Ca^2+^ and K^+^ currents were blocked by using the same solutions that we used for the voltage-clamp recordings.

### Drugs

Chemicals and drugs were purchased from Sigma. PHT was dissolved in dimethylsulfoxide (DMSO), stored at −20°C and the day of the experiment added to the external solution at final concentrations (DMSO was always <1‰), which were tested perfusing the whole bath of the recording chamber. PHT effects were tested 2, 4, 6, 8 and 10 min after the beginning of the drug perfusion; the measurements included in the analysis were obtained from the recordings made after at least 6–8 min. PHT washout was reached after 20 min of perfusion with control extracellular solution. Control experiments were made by perfusing the slices with the external solution alone (4 neurons) or with external solution and DMSO at the same final concentration for the experiments with PHT (8 neurons), which showed that the observed effects were PHT-specific.

### Data analysis

The data were analyzed using pClamp8 (MDS-Axon Instr.) and Origin 7.5 (Origin Lab.). Conductance-voltage (g-V) relationships (activation curves) were calculated from the currents recorded applying voltage ramps using the equation g = I_Na_/(V–E_Na_), in which I_Na_ is the recorded Na^+^ current measured at potential V, and E_Na_ the calculated equilibrium potential. The normalized activation curves were fit to Boltzmann relationships in the form G/G_max_ = 1/{1+exp[(V½–V)/k]}, where G_max_ is the maximal peak conductance, G the peak conductance at each test voltage, V_½_ the voltage at which half-maximal activation is reached, and k the slope factor. The steady-state inactivation curves were fit to Boltzmann relationships in the form: I/I_max_ = 1/{1+exp[(V_½_-V)/k]}+b, where I/I_max_ is the relative current, V_½_ the voltage of half-maximal inactivation, k the slope factor and b the baseline. The development of inactivation was evaluated by fitting the data points with bi-exponential functions in the form y  =  y_0_ + A_1_ exp(-t/τ_1_) + A_2_ exp-(t/τ_2_)+baseline. Simulation curves of inactivation kinetics were generated with tri-exponential functions, adding a time constant and imposing the values of the parameters; they were then fit with bi-exponential functions as for the experimental data. Dose response curves were fit with rectangular hyperbolas. Fits were achieved using the Levenberg – Marquardt algorithm with Origin 7.5.

The data are given as mean values ± SEM, and were statistically analyzed using paired t-test or ANOVA (the logarithmic transformation was used when data were compared as ratios); non-parametric tests (Wilcoxon or Kruskal-Wallis) were also used for comparison and gave similar results.

## Results

We recorded I_NaP_ with the whole-cell configuration of the patch-clamp technique in neocortical layer V or layer II/III pyramidal neurons in somatosensory cortex slices. The slice preparation allows the study of identified subtypes of neurons and the maintenance of a physiological cell background. I_NaP_ can be elicited by both voltage steps and ramp depolarizations; slow depolarizing voltage ramps are often used because they inactivate I_NaT_ allowing the selective recording of I_NaP_ and an evaluation of its voltage dependence of activation [1], [19]. We generally used depolarizing voltage ramp stimuli (from −70 to +20 mV) with a slope of 50 mV s^−1^ (ramp duration 1.8 s), whereas ramps with a different slope or depolarizing steps were used in a subset of experiments. I_NaP_ began to activate between −60 and −50 mV, and peaked between −40 and −35 mV (Figure 1A). In the presence of 1 µM TTX, the inward current was completely abolished and the ramp protocols evoked only a small outward current that activated at membrane potentials more positive than −35 mV (Figure 1A), which was similar to the current characterized by [45]. The peak of I_NaP_ was unaffected by this small TTX-insensitive outward current that activated at more positive potentials.

I_NaP_ undergoes a process of inactivation and we evaluated its kinetics by applying inactivating prepulses before the depolarizing voltage ramp. As we have previously described [19], the kinetics of inactivation was biphasic and could be well fit by bi-exponential functions (Table 1). The curve of the development of I_NaP_ inactivation obtained using TTX-subtracted traces completely overlapped with that obtained using traces that were not TTX-subtracted (Figure 1B and 1C). In order to better evaluate the effect of PHT, in most of the experiments we used a concentration of 100 µM, which is above the clinical range. However, in a subset of experiments we used concentrations in the clinical range (as low as 5 μM).

**Table 1:** Parameters of activation, voltage-dependence and time-dependent inactivation under control conditions and in the presence of PHT.

		Control	PHT 100μM
**Activation** (n = 21; *LV and LII-III pooled*)			
	V½ (mV)	−43.6±0.9	−44.6±1.2
	K (mV)	3.1±0.1	3.2±0.2
**Steady state inactivation** (n = 6, *pooled*))			
	V½ (mV)	−35.3±1.1	−44.3±1.3**
	K (mV)	9.1±1.2	7.3±0.5
**Time dependent inactivation**			
Layer V at -20mV(n = 10)	τ_1_ (ms)	680±120	330±50**
	Aτ_1_ (%)	28±3	29±4
	τ_2_ (ms)	9000±1500	6800±800*
	Aτ_2_ (%)	44±4	45±4
	Baseline (%)	27±3	25±4
Layer V at +40mV (n = 9)	τ_1_ (ms)	167±24	115±20*
	Aτ_1_ (%)	30±4	39±5
	τ_2_ (ms)	4873±375	1850±321*
	Aτ_2_ (%)	54±6	45±5
	Baseline (%)	16±3	16±4
Layer II-III at -20mV (n = 8)	τ_1_ (ms)	350±70	125±40*
	Aτ_1_ (%)	28±2	31±3
	τ_2_ (ms)	8800±500	4800±600*
	Aτ_2_ (%)	45±3	43±4
	Baseline (%)	24±2	23±3

* = p<0.05;** =  p<0.01 (paired t-test). These values have not been corrected for the junction potential error (see Methods).

### Effect of PHT on I_NaP_ activation and peak amplitude

The voltage dependence of I_NaP_ activation was studied as in Aracri et al. (2006). Because we observed no significant difference in its properties among neurons belonging to different layers, we pooled the data from layer V and layer II/III. The properties of the voltage dependence of activation were not modified by the application of 100 µM PHT in any of the tested neurons (not shown in the figures); the mean values of the parameters of the Boltzmann fits of the activation curves are shows in Table 1.

**Figure 2:**
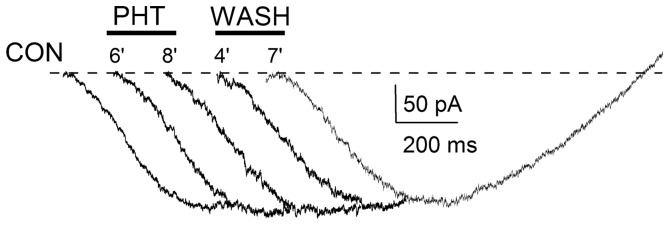
I_NaP_ recorded in a representative pyramidal neuron with 50 mVs^−1^ voltage ramps. Current traces are shown under control conditions, in the presence of 100 µM PHT and during PHT washout.

Strikingly, the peak amplitude of I_NaP_ after application of 100 µM PHT was not significantly different from that measured under control conditions (Figure 2). In fact, the mean peak amplitude was −258±28 pA under control conditions and −278±31 pA in the presence of 100 µM PHT. In a subgroup of 9 neurons we were able to evaluate I_NaP_ amplitude also after PHT washout, but again we did not observe any effect: the peak amplitude was −362±79 pA under control conditions, −373±69 pA in the presence of PHT, and −337±74 pA after PHT washout.

### Effect of PHT on the voltage-dependence of I_NaP_ inactivation

**Figure 3:**
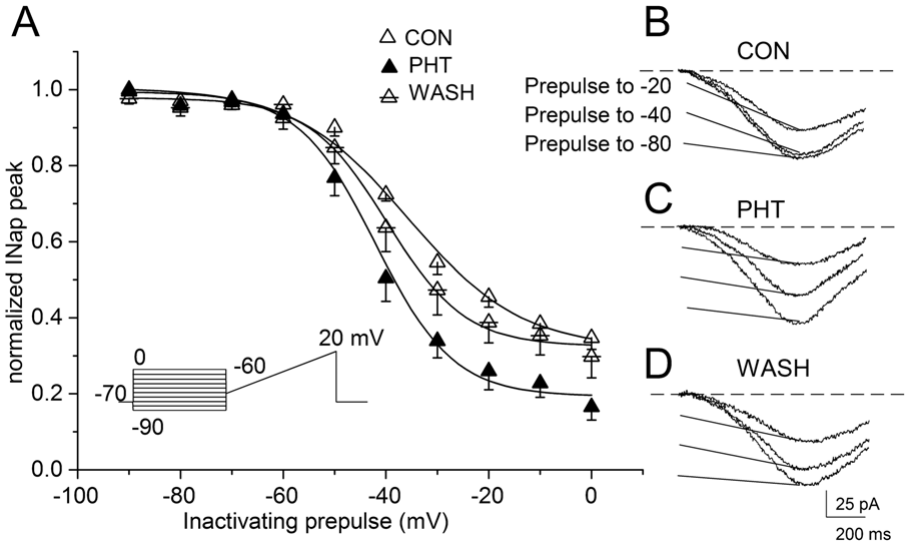
Effect of PHT on the voltage-dependence of I_NaP_ inactivation. A, effect of 100 µM PHT on the activation curve; the inset shows the stimulus protocol (prepulses were 10 s in duration; ramps had a slope of 50 mV s^−1^). Panels B, C and D show the current peaks evoked after different prepulses in a representative neuron. In the presence of PHT, the currents evoked after inactivating prepulses at −40 and −20 mV are clearly reduced with respect to those evoked under control conditions, and partially recover during PHT washout.

We evaluated the effect of 100 µM PHT on the voltage-dependence of I_NaP_ inactivation in layer V neurons using 10s inactivating prepulses, which induce a quasi-steady state inactivation (Aracri et al. 2006). In the presence of the drug, the voltage dependence of inactivation was significantly shifted towards more negative potentials by 7.0±1.4 mV (p<0.007; Figure 3; Table 1) without modifications of the slope. Because of the shift of the curve, under control conditions the I_NaP_ peak began to be significantly reduced by inactivating prepulses to −40 mV (ANOVA test, p<0.0001), whereas in the presence of PHT it began to be significantly reduced by inactivating prepulses to −50 mV (p<0.003). Therefore, PHT has an effect on I_NaP_ inactivation.

### Effect of PHT on the development of I_NaP_ inactivation

**Figure 4:**
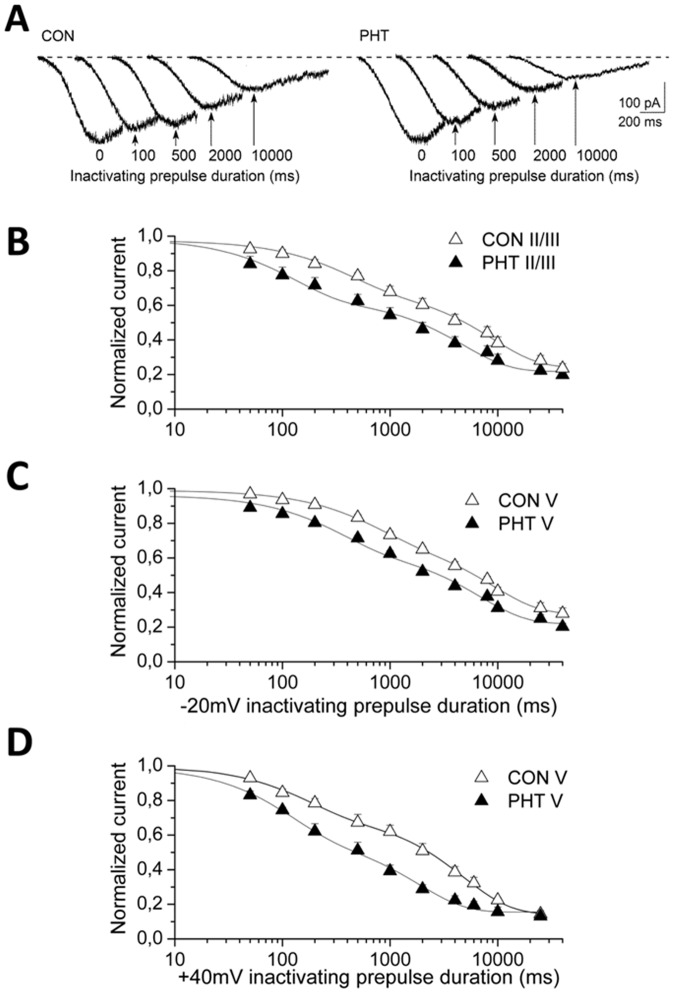
Effect of PHT on the development of I_NaP_ inactivation. Effect of 100 µM PHT on I_NaP_ evoked without (leftmost traces) and with inactivating prepulses to −20 mV lasting from 100 ms to 10 seconds in a representative layer V neuron. The arrows indicate the peak current evoked using depolarizing ramp stimuli under control conditions (left) and in the presence of 100 µM PHT (right). Development of I_NaP_ inactivation in layers II/III (B) and V (C) at −20 mV and in layer V at +40 mV (D) on a semi-logarithmic scale, under control conditions (open triangles) and in the presence of 100 PHT µM (black triangles); the data points were fit to bi-exponential functions with a baseline (see Table 1).

We evaluated the effect of 100 µM PHT on I_NaP_ evoked with our standard voltage ramp without inactivating prepulse and at the end of inactivating prepulses to −20 mV. Figure 4A shows representative recordings of different selected prepulse durations (100 ms, 500 ms, 2 s and 10 s) from a layer V neuron in control and with PHT. Notably, PHT was able to reduce I_NaP_ only when it was inactivated by the prepulse. Semi-logarithmic plots of the mean development of inactivation are shown in Figure 4B and 4C for prepulses to −20 mV and in Figure 4D for prepulses to +40 mV. The data at −20 mV provides some information on the effect of PHT on I_NaP_ inactivation at potentials that are typical of the depolarized plateaux of cortical neurons (see below), but the observed effect at this potential depends both on the shift of the voltage dependence of inactivation (Figure 3) and on actual modifications of the kinetics of the development of inactivation. The data at +40 mV provides a quantification of the time course that does not depend on the shift of the voltage dependence of inactivation [46]. Confirming our previous results [19], the development was well fit to the sum of two exponential relationships and was quantitatively different between layer V and layer II/III pyramidal neurons (Figure 4B and 4C). In fact (Table 1), the faster time-constant describing the time-dependent inactivation was significantly slower in the pyramidal neurons of layer V than in those of layer II/III (680±120 vs. 350±70 ms at −20 mV; p<0.011) and the slower time-constant was not significantly different (9.0±1.5 s, layer V; 8.8±0.5 s layer II/III, at −20 mV); a small current remained in all of the neurons, even after the longest inactivating prepulses, without any difference between the two layers (27±3% and 24±2% of the unconditioned current peak, in layer V and layer II/III respectively). The perfusion with 100 μM PHT significantly accelerated both the first and the second time-constant describing I_NaP_ inactivation in all of the tested neurons (layer II/III n = 8: p<0.020 and p<0.023; layer V n = 10: p<0.002 and p<0.019). The mean values of the parameters of the exponential fits are shown in Table 1.

Because of the accelerated time-dependent I_NaP_ inactivation, the amplitude of I_NaP_ evoked after conditioning prepulses lasting longer than 50 ms was significantly reduced in the presence of 100 µM PHT in comparison with that measured under control conditions in both layers II/III and layer V neurons. In fact, after a 100 ms conditioning prepulse the peak of I_NaP_ was reduced by 15.3±1.8% with PHT and 11.4±4.0% in control (p<0.005 and p<0.001); after a 500 ms depolarizing prepulse, it was reduced by 19.8±4.4% with PHT and 13.9±3.1% in control (p<0.002). In both layers, the “really persistent” non-inactivating current remaining after the longest inactivating prepulses (40 s) was not modified by PHT (baseline in the plots of Figure 4B and 4C, see Table 1).

We evaluated the effect of PHT on the kinetics of development of inactivation at +40 mV only in layer V pyramidal neurons, because the application of long pulses at positive potentials makes these experiments particularly challenging. Figure 4D shows the curves of the development at +40 mV in control and after application of PHT, which were well fit to a double exponential. PHT accelerated both the time constants. In fact, the faster time-constant was 167±37 in control and 107±14 ms with PHT (n = 7; p = 0.04) and the second time-constant was 4.9±0.8 s in control and 2.1±0.2 s with PHT (p<0.01). These results show that PHT is able to induce an acceleration of the development of inactivation independently from the shift of the voltage dependence of inactivation.

### Dose-response relationship of PHT effect

In order to better characterize the action of PHT and examine its effect at lower concentrations in the therapeutic range, we studied the dose-response relationship of I_NaP_ inhibition. As the inhibitory effect of 100 µM PHT was substantially similar in layers II/III and V, we evaluated the effects of different PHT concentrations by testing the I_NaP_ evoked in 29 layer V neurons after inactivating prepulses of 200 and 500 ms. We selected these inactivating prepulses in order to shorten the very long protocols needed to evaluate the whole time-course of I_NaP_ inactivation, and considering that the inhibitory effect of PHT on I_NaP_ evoked after inactivating prepulses of the selected durations was consistent and statistically significant.

**Figure 5:**
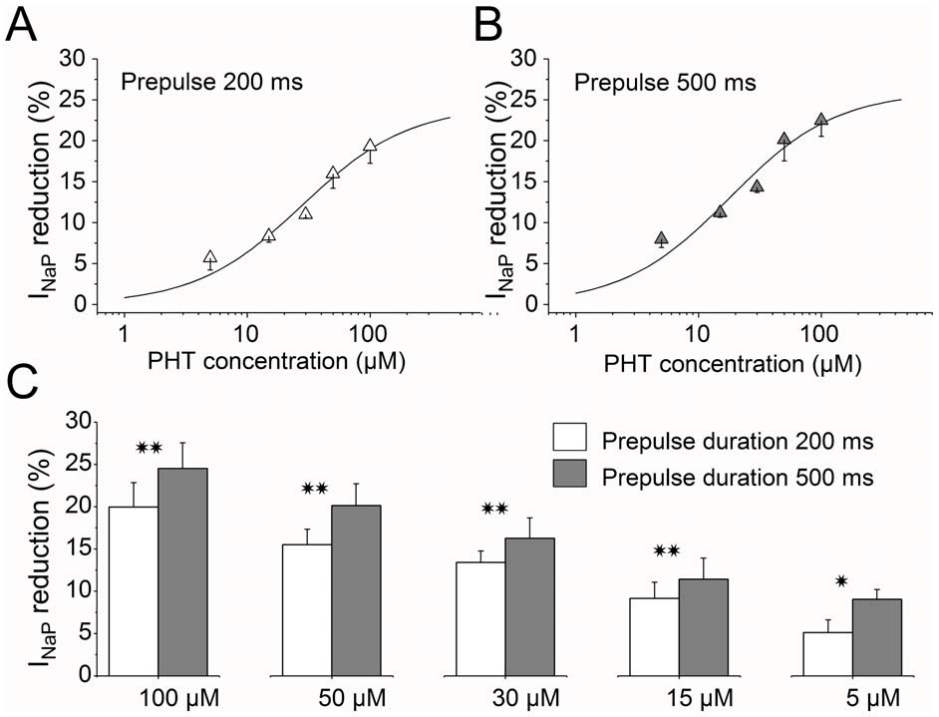
Effect of different concentrations of PHT on I_NaP_ evoked using inactivating prepulses of different durations. Panels A and B show the dose response curves with prepulses of 200 ms (A) or 500 ms (B); higher concentrations of PHT have not been used because of its solubility limits, and the maximal block was not reached. The solid lines are fit to rectangular hyperbolas that gave apparent IC_50_ values of 28 and 18 µM and apparent maximal block of 24 and 26% respectively. All of the PHT concentrations used induced a statistically significant reduction of I_NaP_ peak amplitude with both 200 and 500 ms inactivating prepulses, as shown in C (**p<0.01; *** = p<0.001; ANOVA test).

The dose-response relationships obtained with PHT concentrations of 5 (n = 7), 15 (n = 5), 30 (n = 8), 50 (n = 8) and 100 µM (n = 10) are displayed in Figure 5. Higher concentrations of PHT have not been used because of its solubility limits. Thus, although the maximal block was not reached and dose-response parameters could not be correctly estimated, experimental data could be fit by a one-to-one binding curve that gave apparent IC_50_ values of 28 µM and 18 µM for inactivating prepulses of 200 and 500 ms respectively (Figure 5A and 5B). Thus, a longer prepulse induced a decrease in the apparent IC_50_. All of the PHT concentrations tested significantly reduced the I_NaP_ peak amplitude evoked after prepulses of 200 and 500 ms (p values varying from <0.04 to <0.0001, ANOVA) (Figure 5C). Conversely, as found in our other experiments, I_NaP_ peak amplitude evoked without inactivating prepulses was similar under control conditions and in the presence of PHT, irrespectively of the concentration of PHT used.

Therefore, these results confirm that PHT reduced I_NaP_ only in conditions in which I_NaP_ underwent inactivation; in these conditions even low concentrations well within the therapeutic range induced a significant reduction. Notably, long lasting depolarizations that can potentiate the effect of PHT on I_NaP_ are central in the pathomechanisms of several neurologic diseases [20], [21], [47].

### PHT blockade of I_NaP_ during slow-ramp stimuli and long depolarizing steps

The lack of any effects of PHT on I_NaP_ that we have observed evoking I_NaP_ by applying depolarizing ramps without inactivating prepulses, contrasts with the data obtained by other authors. For example, PHT was able to reduce I_NaP_ amplitude in single electrode voltage clamp recordings from layer V pyramidal neurons in cortical slices [37]. However, in those experiments I_NaP_ was evoked applying much slower depolarizing voltage ramps (10 mV s^−1^) than our standard 50 mV s^−1^ ramp, and this slow stimulation can be expected to induce significant I_NaP_ inactivation. Therefore, we hypothesized that the slope of the depolarizing ramp can be an important parameter in the assessment of PHT action.

**Figure 6:**
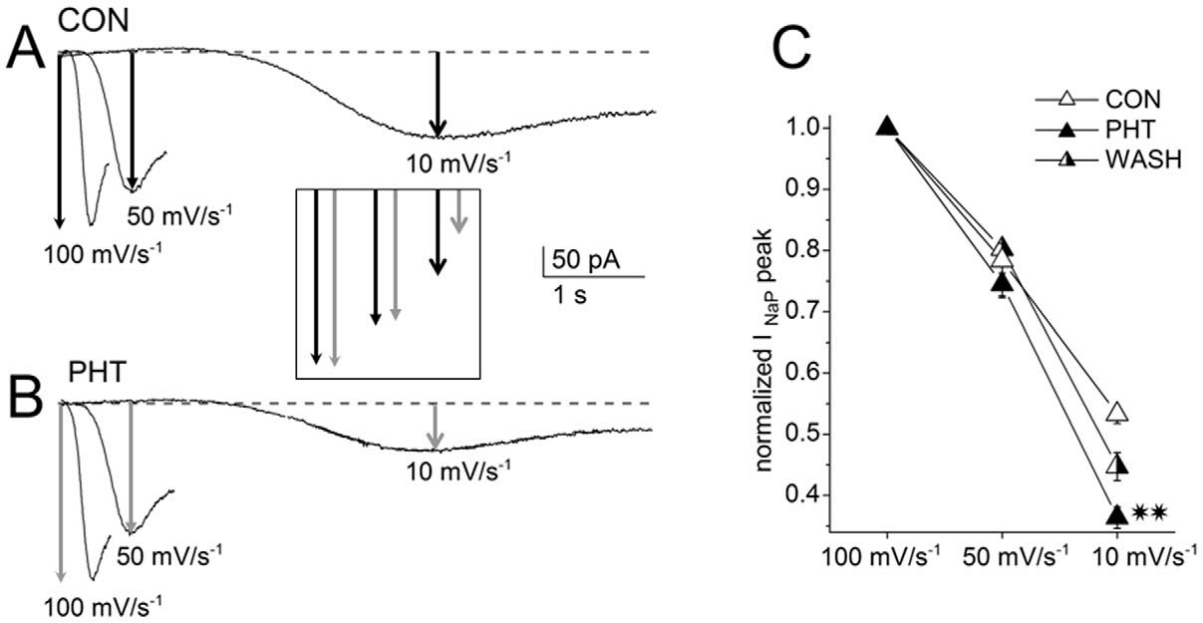
I_NaP_ evoked by ramps with different slopes. Current traces are shown under control conditions (A) and in the presence of PHT (100 µM) (B). The amplitude of the current decreased with the duration of the ramps and the inhibitory effect of phenytoin became clear and significant for I_NaP_ evoked by slower ramps (arrows). The inset shows a graphical comparison of I_NaP_ amplitude in the different conditions. The graph in C displays the mean values of the I_NaP_ peaks measured on five neurons in response to 100, 50 and 10 mVs^−1^ ramps under control conditions, in the presence of PHT and after PHT wash out.

In order to test this hypothesis, we investigated the effect of the slope of the ramp on the amplitude of the peak of I_NaP_ in control and on the action of 100 µM PHT, using voltage ramps of 10, 50 and 100 mV s^−1^. Indeed, I_NaP_ peak amplitude and PHT effect were modulated by the slope of the ramp. When I_NaP_ was evoked with 50 mV s^−1^ ramps, its peak amplitude was reduced by about 20% with respect to 100 mV s^−1^ ramps both under control conditions and in the presence of PHT (18.1±2.5% in control and 22.1±1.1% with PHT, n = 5; Figure 6A and 6B). Very slow ramps of 10 mV s^−1^ induced a more pronounced reduction under control conditions (47.1±1.9% in comparison with ramps of 100 mV s^−1^) and, strikingly, the reduction was significantly larger in the presence of PHT (65.3±1.3%, p<0.001; paired t-test) (Figure 6C). These data show that inactivation of I_NaP_ is induced by slow depolarizing ramps and that the effect of PHT is inversely proportional to the slope of the ramps, depending therefore on the inactivation of I_NaP_ induced by the stimulation. Notably, this effect does not depend only on the intrinsic slow binding of PHT, because in our experiments an inactivating prepulse as short as some tens of ms applied before the 50 mV s^−1^ ramp could induce PHT-dependent I_NaP_ inhibition (Figures 4 and 5). Thus, the fact that the effect of PHT on I_NaP_ was evident only when the current was elicited with very slow ramps or with standard ramps preceded by inactivating prepulses and the lack of effect on the non-inactivating component of the current even after 40s of depolarization (see above and Figure 4) suggest that PHT does not effectively block the open conformation of the channel.

**Figure 7:**
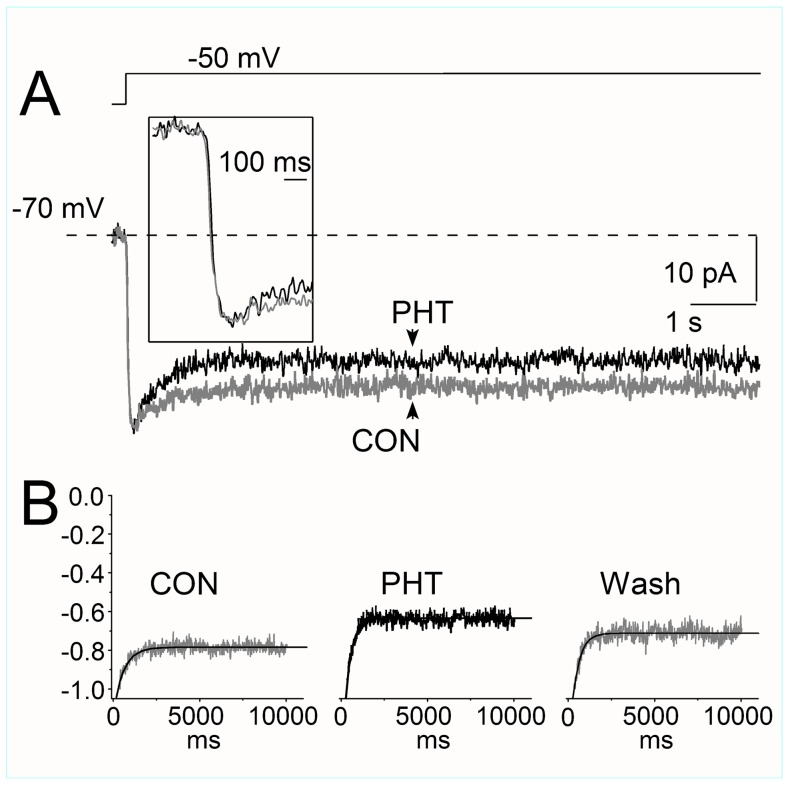
I_NaP_ evoked by a 10 s step stimulus to −50 mV. Current traces are shown under control conditions (gray line) and in the presence of 100 µM PHT (black line) in a representative layer V neuron (A); note that the amplitude of the current in the first few hundred milliseconds of the trace is unaffected by PHT (inset in A). The average of the currents obtained in five layer V neurons and normalized to the maximum (B) shows that the decay time constant describing the current inactivation is accelerated and enhanced in the presence of PHT (middle panel). The decay was fit with a mono-exponential relationship (tick line); Note that in these experiments the effect of PHT is particularly small because at −50 mV I_NaP_ inactivation is minimal. See text for details.

We confirmed these observations by evoking I_NaP_ in layer V neurons with long (10 s) depolarizing voltage steps to −50 mV, from an holding potential of −70 mV (Figure 7). I_NaP_ does not undergo inactivation at the potential of −70 mV (Figure 3). At the test potential of −50 mV I_NaP_ is small (about 20% of the maximum), but I_NaT_ is basically absent and some I_NaP_ inactivation develops during the pulse (see Figure 3 and [19]). Therefore, with this stimulus it is possible to record non-inactivated I_NaP_ in the first tens of milliseconds of the test pulse without a substantial contamination of poorly clamped I_NaT_, and then to follow the development of its inactivation. Figure 7 shows traces recorded with this long depolarizing pulse in which, under control conditions, there was about 20% inactivation at the end of the pulse, consistently with the data obtained with depolarizing ramps in which inactivation at this potential was about 10–15%; (Figure 3 and Aracri et al. 2006). The decay of the current during the depolarization could be fit to a mono-exponential relationship whose time constant was 580±30 ms, which is again similar to the faster time constant obtained by evoking I_NaP_ with ramps (Figure 4, Table 1). The slower phase of inactivation, which is evident in Figure 4, was not well resolved in these recordings, probably because the percentage of inactivation is small at this test potential and the development was limited to 10 s. Remarkably, application of PHT did not induce a reduction of I_NaP_ at the beginning of the pulse, but its action developed slowly during the depolarization leading to a decay of I_NaP_ characterized by a time constant of 395±15 ms, which is similar to that obtained applying ramps preceded by inactivating steps in the presence of PHT (see Figure 4, Table 1). Upon wash out of PHT, both the amplitude of I_NaP_ at the end of the 10 s pulse and the kinetics of the decay during the pulse recovered to values similar to those of control conditions (τ = 480±18 ms). Notably, the effect of PHT was smaller in this experiment because at −50 mV I_NaP_ inactivation is not pronounced.

Therefore, we have not observed a fast block of I_NaP_ at the beginning of depolarizing steps, in which channels should predominantly be in the open/non-inactivated conformation. In fact, the action of PHT developed slowly during the depolarization. This property is shared by the process of inactivation of I_NaP_, but the kinetics of the block by PHT was faster than the development of I_NaP_ inactivation in control, consistent with the data obtained with depolarizing ramps, and faster than the suggested PHT intrinsic binding kinetics [48], [49].

### Effect of PHT on recovery from I_NaP_ inactivation

**Figure 8:**
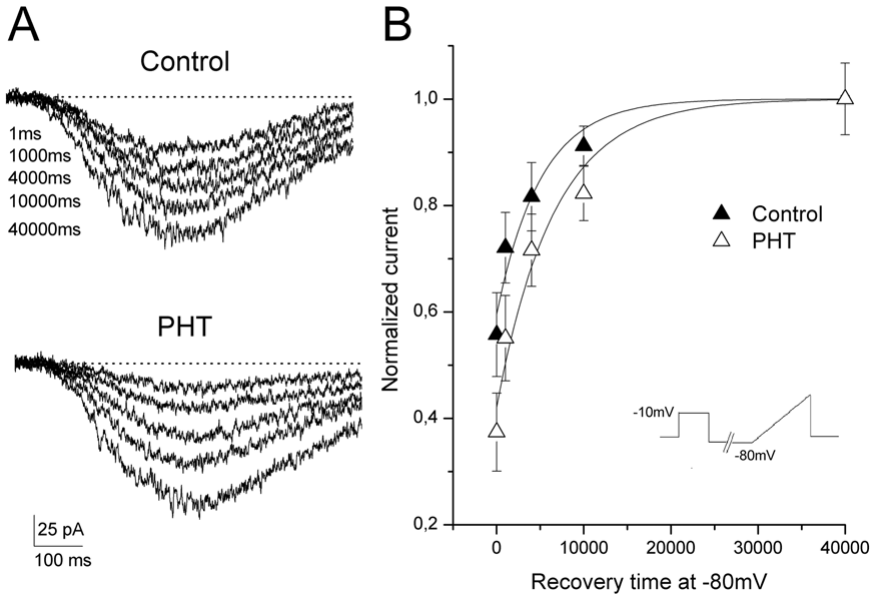
Effect of PHT on recovery from I_NaP_ inactivation. A, representative traces in control (above) and with 100 μM PHT (below) recorded after a 20 s-long inactivating prepulse to −10 mV and a recovery period at −80 mV of 1 ms, 1000 ms, 4000 ms, 10000 ms and 40000 ms (see stimulus in B). B, plot showing average recovery in control (black triangles) and with 100 μM PHT (hollow triangles). The lines are single exponentials obtained averaging the parameters of the fits of the single cells (see text for details).

It has been shown that PHT can slow down the recovery from inactivation of I_NaT_. We tested the effect on the recovery from I_NaP_ inactivation with a stimulus composed of a 20 s-long inactivating prepulse at −10 mV, recovery periods at −80 mV ranging from 1 ms to 40 s in duration, and a 50 mV/s ramp for evoking I_NaP_ (n = 7; Figure 8). Under control conditions the inactivating prepulse induced a reduction of I_NaP_ amplitude to 56±7.8% of the unconditioned value, and the current recovered with a time constant of 5.1±1.2 s. Upon perfusion with 100 μM PHT the prepulse induced a reduction of I_NaP_ amplitude to 37±19% of the unconditioned amplitude, which was significantly different than the reduction observed in control (p = 0.03),. However, the recovery was characterized by a time constant of 6.4±1.3 s, which was not statistically different in comparison with the control. Therefore, recovery from I_NaT_ inactivation was not affected by PHT.

### Effect of PHT in current clamp recordings

**Figure 9:**
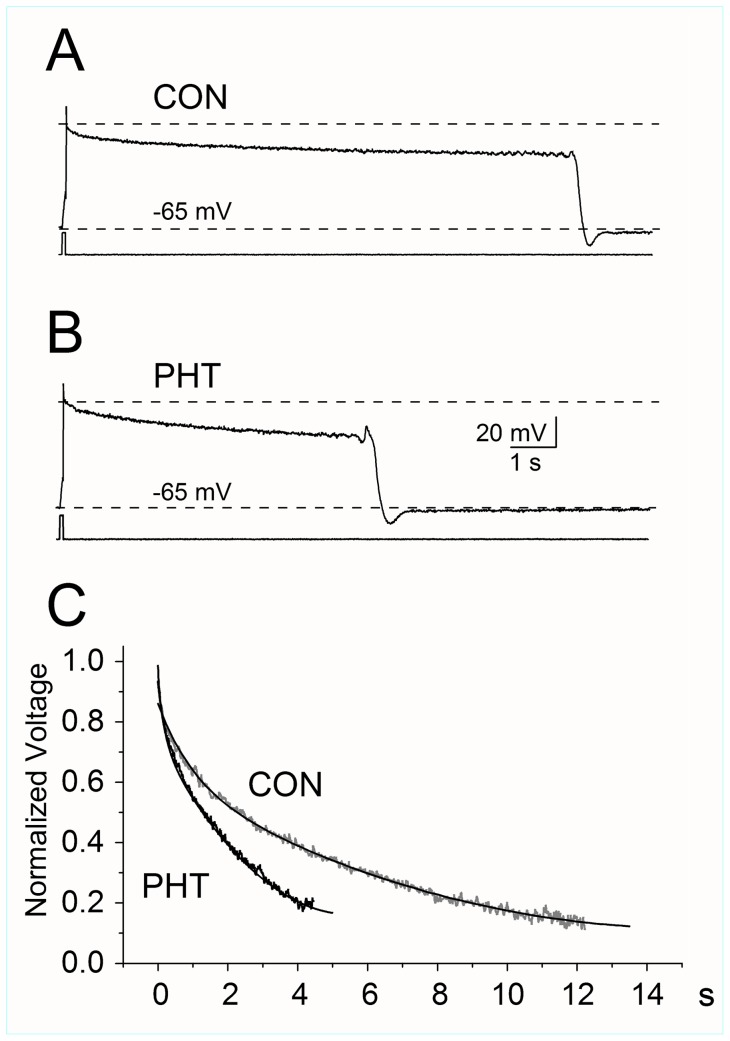
Effect of PHT on depolarized plateau recorded in current clamp. Depolarized plateau induced by the intracellular injection of a brief (40 ms) depolarizing pulse in a representative neuron recorded in current clamp configuration. Long-lasting depolarizations following the action potential were observed in the presence of K^+^ and Ca^2+^ channel blockers and are most likely sustained by the I_NaP_ flowing after the fast inactivation of the transient Na^+^ current. The long depolarized plateau observed under control conditions (A) were shortened in the presence of PHT (100 µM) (B). In five neurons, the decay of the depolarizing plateau was fit by bi-exponential functions; the values of the time-constants were comparable to those describing the time-dependent inactivation of I_NaP_ and were significantly shortened by PHT. Panel C shows the mean plateau potentials normalized to their maximal value and the bi-exponential fitting obtained averaging the parameters of the fits of the single cells.

In order to verify the effect of PHT on the properties of I_NaP_–sustained neuronal depolarizations, we tested five layer V neurons recorded in current clamp configuration. The contribution of I_NaP_ on membrane depolarization was revealed by blocking Ca^2+^ and K^+^ currents, using the same solutions of the voltage-clamp experiments. Under these conditions, the action potential evoked by the intracellular injection of a relatively brief (40 ms) depolarizing pulse was followed by long-lasting plateau potentials, which are most likely sustained by I_NaP_ ([1], [5], [19], [39]. Figure 9 shows the voltage traces of a representative Layer V neuron in control (A) and in the presence of 100 µM PHT (B). The long plateau following the action potentials under control conditions (lasting on average of 11.4±0.6 s) were substantially reduced in the presence of 100 µM PHT (to an average of 5.8±0.6 s; n = 5; p<0.04). The decay of the depolarizing plateau could be fit using a bi-exponential function both in control conditions and after application of PHT (Figure 9C). PHT induced a decrease of the time-constants of the decay that was comparable to that found in the development of I_NaP_ inactivation (τ_1_ = 205±34 ms under control conditions vs. 59±13 ms in the presence of PHT, p<0.03; τ_2_ = 6.7 ±1.2 s, under control conditions vs. 2.3±0.4 s, in the presence of PHT, p<0.02; n = 5). Therefore, the effects observed on I_NaP_ are consistent with those observed on plateau potentials, showing that PHT can actually reduce long lasting depolarizations sustained by I_NaP_.

### Inferences on the mechanism of action

The effects of therapeutic Na^+^ channel blockers have been mainly investigated on I_NaT_ and in general interpreted according to the modulated receptor model [50], in which the drug binds to the channel with different affinities according to the functional state of the channel, stabilizing the state to which it binds. It has been established that the affinity of Na^+^ channel blockers for the closed “resting” states is low, whereas affinity is much higher for “activated” channels (channel states induced by depolarizations or trains of action potentials) [21], [48]. The small effect on resting channels (current reduction after prolonged periods at hyperpolarized potentials) has been interpreted as blockade of the pore through a low affinity receptor site; whereas the effect on activated channels has been related to a high affinity receptor site, although it has been quite difficult to disclose to which state (fast inactivated, slow inactivated or even open) Na^+^ channel blockers actually bind with highest affinity.

**Figure 10:**
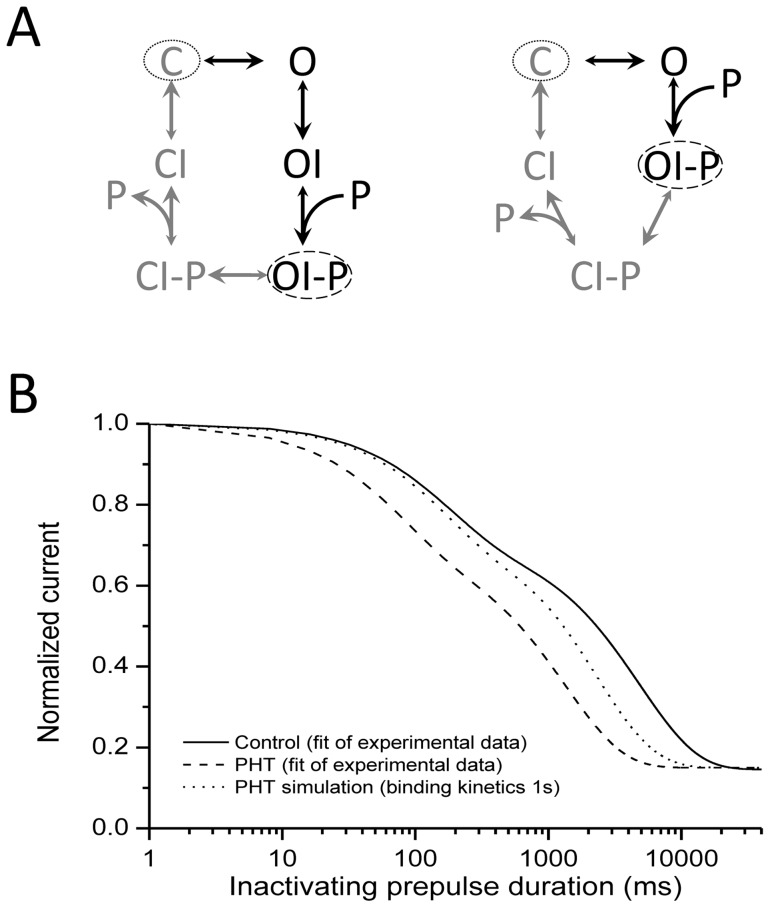
Inferences on the mechanism of action. A; simplified gating schemes illustrating the action of PHT, excluding an interaction with the open state of the channel. The scheme on the left depicts PHT as a pure inactivated state stabilizer that binds to the inactivated state of the channel; the scheme on the right is an interpretation of our results and depicts PHT as an inactivated state stabilizer that binds to the inactivated state and to a hypothetical intermediate in the inactivation pathway. C, O, OI and CI are the closed, open, open-inactivated and closed-inactivated states of the Na^+^ channel; P is PHT, which in both cases has much higher affinity to the I state than to the C (resting) state. The black parts of the schemes are the transitions induced by depolarized potentials: the dimension of the arrowhead indicates the value of the rate constants and the dashed oval indicates the absorbing state at depolarized potentials. The gray parts are the transitions induced by repolarizations and the dotted oval is the absorbing state in these conditions. The main difference between the two schemes is the fact that on the left PHT binds to channels already in the inactivated state stabilizing it, whereas on the right it can also accelerate the kinetics of inactivation by binding to a hypothetical intermediate, similarly to a catalyst in a chemical reaction. B; the simulated curve of development of I_NaP_ inactivation shows that the effect of PHT cannot be obtained with a simple intrinsic slow binding of PHT to inactivated channels as in scheme A, left. The dotted line is a simple simulation of the maximal effect of PHT on development of I_NaP_ inactivation at +40 mV in Layer V neurons according to the scheme in A, left. It is assumed that that the action of PHT is intrinsically slow, developing with a time constant of 1 s (Kuo and Bean, 1994; Kuo et al, 1997), and that it binds irreversibly to inactivated channels. Solid and dashed lines are the fits of the experimental data in control and with PHT respectively, which are shown in Figure 4 and in Table 1. The simulated curve (h_Dt_, development of inactivation in the presence of PHT) has been obtained with the following equation: h_Dt_ = h_t_{1-[(1-h_t_) d_t_]}, in which h_t_ is the curve of development in control (fraction of channels available as a function of time at +40 mV) and d_t_ the fractional binding of PHT (kinetics of PHT binding). It is evident that the simulated curve does not approximate the experimental curve in the presence of PHT (compare the dashed line with the dotted one). In order to quantitatively compare the parameters with those of the experimental curves (Table 1), the simulated curve was fit with a double exponential relationship that gave the following parameters: τ_1_ = 0.12 s, A_1_ = 0.25, τ_2_ = 2.4 s, A_2_ = 0.6, baseline = 0.15, which are different in comparison with those obtained from the experimental curve in the presence of PHT (Table 1). Thus, PHT effect cannot be simulated with a simple intrinsic slow binding of PHT to the channels in the inactivated conformation; an acceleration of the rate constants of development of I_NaP_ inactivation as in scheme A, right, is more consistent with the experimental data (see text).

According to the modulated receptor framework (Figure 10, A left), PHT would bind to Na^+^ channel inactivated states and its slow kinetics of I_NaT_ block has been considered to be related to an intrinsically slow binding of the drug, rather than to a selective effect on slow inactivation, because PHT action develops faster than slow inactivation of I_NaT_ [48], [49]. Similarly, our data show that the effect of PHT develops faster than I_NaP_ inactivation. However, in our experiments the application of PHT induced an acceleration of both the faster and the slower phases of I_NaP_ inactivation (Figure 4), consistent with a direct effect of PHT on the kinetics of I_NaP_ inactivation and not with a pure stabilization of the inactivated state as in the classic modulated receptor model, which would accelerate only the slower phase (Figure 10B). Thus, PHT may accelerate the rate constants of the inactivation process of I_NaP_ by stabilizing a kinetic intermediate in the inactivation pathway; accelerating I_NaP_ inactivation similarly to a catalyst in a chemical reaction (Figure 10A, right). In Figure 10, the PHT bound open-inactivated state is depicted as the absorbing state at depolarized potentials. Considering a single homogeneous population of channels, this would lead to a complete blockade of I_NaP_, inconsistent with our results showing that the non-inactivating component is resistant to PHT action and that the relative amplitudes of the exponential phases of the development of inactivation are not modified by PHT (see Table 1 and Figure 10). An alternative hypothesis more consistent with our data would be the existence of three distinct populations of channels, two of them inactivating with different time constants and sensitive to PHT, the third one non-inactivating and insensitive to PHT (the schemes of Figure 10 refer to a single population of inactivating channels). The lack of effect on recovery from I_NaP_ inactivation shows that unbinding of PHT during transitions from inactivated to closed states is not a rate limiting step.

## Discussion and Conclusions

Because I_NaP_ inactivation has been in general overlooked, the effects of Na^+^ channel blockers on the properties of I_NaP_ inactivation have not been previously investigated, and an open channel block has generally been implied [21]. Consistently with our previous results [19], we have observed I_NaP_ inactivation in both layer V and Layer II/III neocortical pyramidal neurons; its kinetics of development was biphasic, leading to a time-dependent decay that was described by a double-exponential function. Moreover, in addition to the slowly inactivating main component of I_NaP_, we observed an apparently “really persistent” non-inactivating fraction of the current that did not inactivate even upon application of very long depolarizing prepulses. This “non-inactivating” I_NaP_ fraction is not present in the entorhinal cortex [18] but has been observed in other brain areas [7], [8].

Remarkably, we have found that I_NaP_ was reduced by PHT only in conditions in which there was substantial I_NaP_ inactivation. In fact, the reduction was observed only when very slow ramps or inactivating pulses were applied; PHT induced an acceleration of both the phases of the development of I_NaP_ inactivation, and a negative shift of the voltage-dependence of inactivation. Interestingly, I_NaP_ inactivation has been observed in different types of neurons, although its properties have been characterized only in few studies. Notably, PHT did not substantially affect the apparently “persistent” non-inactivating I_NaP_ component remaining even at the end of the longest (40 s) inactivating prepulse.

In theory, because of the complex and heterogeneous morphology of cortical pyramidal neurons, the properties of the time-dependent decay of I_NaP_ in our slice recordings might be influenced by space-clamp issues. However, we found that, differently from inactivation, the properties of I_NaP_ activation were similar in neurons recorded in different cortical layers and thus with different electrotonic properties [19], and they were not modified by PHT. These findings suggest that the properties of I_NaP_ inactivation observed under control conditions and the modifications induced by PHT can be considered specific and real.

The inactivation process of I_NaP_ is phenomenologically similar to the slow inactivation of I_NaT_ [51], [52], but much less is known about its characteristics, and it remains to be elucidated whether at the molecular level it is generated by the same conformational changes that produce I_NaT_ slow inactivation. The effect of PHT on I_NaT_ slow inactivation has been investigated in other studies [48], [53]., Moreover, the effects of PHT that we have observed on I_NaP_ are similar to those reported for inhibition of I_NaT_ by the novel antiepileptic drug lacosamide, which selectively enhances I_NaT_ slow inactivation without modifying fast inactivation and recovery from slow inactivation, and might bind to a novel drug binding site that is distinct from the binding site responsible for enhancement of fast inactivation [54]. However, lacosamide has only been tested on I_NaT_; to our knowledge, our study is the first one showing that a Na^+^ channel blocker inhibits I_NaP_ acting selectively on its inactivation properties. Moreover, our results are inconsistent with open channel block and suggest that the acceleration of the development of I_NaP_ inactivation is a modification of its kinetic properties: data obtained with prepulses to +40 mV indicate that PHT inhibition of I_NaP_ does not depend on the negative shift of the voltage dependence of inactivation.

As already highlighted, we did not find any significant reduction in I_NaP_ when it was evoked with our standard ramp in the absence of an inactivating prepulse. Notably, this lack of effect in the absence of inactivating pre-pulses contrasts with some previous findings indicating that PHT was capable of reducing I_NaP_ without inactivating prepulses [35]–[38]. However, these discrepancies may be due to different experimental conditions. For instance, Lample et al. [37] evoked I_NaP_ with very long and shallow depolarizing ramps, which can induce substantial I_NaP_ inactivation. Thus, the effect observed on I_NaP_ amplitude may have been due to the PHT-induced acceleration of I_NaP_ inactivation, which we have disclosed in our study. Niespodziany et al. [38] evaluated the effect of PHT on I_NaP_ in the CA1 area of hippocampal slices after a perfusion lasting more than 20 minutes. Differently, we began to test I_NaP_ 6–8 minutes after the onset of PHT perfusion, a duration that was however about 3-fold longer with respect to that needed for TTX to completely block Na^+^ currents and sufficient for PHT to consistently modify the properties of inactivation. Moreover, we did not find consistent changes on I_NaP_ evoked without inactivating prepulses during perfusions lasting up to 15 min or during the washouts. We cannot exclude that very long perfusions result in higher local concentrations, leading to an effect also on I_NaP_ peak amplitude evoked without inactivating prepulses. However, some of the discrepancies that we have described above may be caused by differences in the properties of I_NaP_ inactivation in different neurons and brain areas. For example, a faster development of inactivation would induce an effect of PHT with faster ramps or with no inactivating prepulses, as well as a leftward shift of the voltage dependence of inactivation that would induce significant I_NaP_ inactivation at the holding potential. It will be interesting to study the properties of I_NaP_ inactivation and to test the effect of PHT in other neurons and brain areas.

The lack of PHT effect on the channels in the open conformation (e.g. the non-inactivating “really persistent” component) could be explained by a low affinity for the open conformation and/or by kinetics constraints. In fact, single channel recordings have shown that channel openings generating I_NaP_ are in general short, with average durations of about 3 and 21 ms in the entorhinal cortex [18] or of about 0.3 and 2–3 ms in other experimental settings [36], [55], whereas longer single openings or long bursts of openings are quite rare. Channels that do not undergo inactivation and generate the “really persistent” component in our experiments could similarly show brief openings and be characterized by low open probability; PHT binding could be too slow to effectively affect channels in the open conformation (thus during the brief openings). Interestingly, Segal and Douglas [36] tested PHT on cultured hippocampal neurons in conditions that induce chronic hyperexcitability, showing that PHT can reduce I_NaP_ in single channel recordings. They observed a decreased number of channel openings, with small effects on the duration of the openings and no effects on open channel current amplitude, consistently with an effect of PHT on the inactivated state of the channel, as we propose here.

It will be interesting to compare in future studies the mechanism of PHT-induced I_NaP_ reduction with that of other Na^+^ channel blockers; this would be important not only for a better insight on mechanisms of action, but also for a clearer understanding of which pathological dysfunction can be better reduced by a specific Na^+^ channel blocker. For instance, I_NaP_ can induce alterations of firing properties, which are probably more involved in epilepsy [2], or very long depolarizations, possibly implicated in cortical spreading depression or intracellular Na^+^ overload with consequent neuronal damage, which are probably more involved in migraine, hypoxic damage and other neurological diseases [20], [33], [47], [56]. The facilitation of I_NaP_ inactivation could reduce pathological depolarizations, leaving largely unaltered shorter I_NaP_ actions. However, in brain regions or pathological states in which the non-inactivating “really persistent” I_NaP_ component is present, a drug that could block also this component would probably have a larger effect than PHT on reduction of long lasting depolarizations and intracellular Na^+^ overload. Interestingly, in some types of neurons this non-inactivating I_NaP_ fraction has not been observed [18], and PHT may have in these neurons more effect on I_NaP_ functions than in others. The similar effects of PHT on neurons in layers II/III and V suggest that, despite the quantitative differences in the properties of I_NaP_ inactivation in different cortical layers [19], this mechanism of I_NaP_ reduction can be widespread in pyramidal cortical neurons.

For better disclosing the mechanism of action, we used in most of the experiments a PHT concentration of 100 μM, which is higher than therapeutic ones. Nevertheless, we found that also lower concentrations (i.e. between 5 and 30 µM), which are more similar to those used in the clinical practice, can induce a significant effect. Moreover, the effect was inversely proportional to the duration of the depolarizing prepulse, showing that in the presence of sustained depolarizations, which are typical of several pathologies [20], [21], [47], lower concentrations of PHT can have a larger effect.

These results help to clarify the range of efficacy and the limits of PHT as an I_NaP_ inhibitor, which should be more effective on I_NaP_–induced long lasting depolarizations than on shorter I_NaP_ actions.
